# A Single Short ‘Tone Burst’ Results in Optimal Drug Delivery to Tumours Using Ultrasound-Triggered Therapeutic Microbubbles

**DOI:** 10.3390/pharmaceutics14030622

**Published:** 2022-03-11

**Authors:** Nicola Ingram, Laura E. McVeigh, Radwa H. Abou-Saleh, Damien V. B. Batchelor, Paul M. Loadman, James R. McLaughlan, Alexander F. Markham, Stephen D. Evans, P. Louise Coletta

**Affiliations:** 1 Leeds Institute of Medical Research, Faculty of Medicine and Health, St James’s University Hospital, Beckett Street, Leeds LS9 7TF, UK; 20mcveigh@gmail.com (L.E.M.); j.r.mclaughlan@leeds.ac.uk (J.R.M.); a.f.markham@leeds.ac.uk (A.F.M.); 2 School of Physics and Astronomy, University of Leeds, Leeds LS2 9JT, UK; r.h.saleh@gu.edu.eg (R.H.A.-S.); py13db@leeds.ac.uk (D.V.B.B.); s.d.evans@leeds.ac.uk (S.D.E.); 3 Nanoscience and Technology Group, Faculty of Science, Galala University, Galala 43711, Egypt; 4 Department of Physics, Mansoura University, Mansoura 35516, Egypt; 5 Institute of Cancer Therapeutics, University of Bradford, Bradford BD7 1DP, UK; p.m.loadman@bradford.ac.uk; 6 School of Electronic and Electrical Engineering, University of Leeds, Leeds LS2 9JT, UK

**Keywords:** ultrasound, triggered delivery, liposomes, chemotherapy, VEGFR2, chirp, tone

## Abstract

Advanced drug delivery systems, such as ultrasound-mediated drug delivery, show great promise for increasing the therapeutic index. Improvements in delivery by altering the ultrasound parameters have been studied heavily in vitro but relatively little in vivo. Here, the same therapeutic microbubble and tumour type are used to determine whether altering ultrasound parameters can improve drug delivery. Liposomes were loaded with SN38 and attached via avidin: biotin linkages to microbubbles. The whole structure was targeted to the tumour vasculature by the addition of anti-vascular endothelial growth factor receptor 2 antibodies. Tumour drug delivery and metabolism were quantified in SW480 xenografts after application of an ultrasound trigger to the tumour region. Increasing the trigger duration from 5 s to 2 min or increasing the number of 5 s triggers did not improve drug delivery, nor did changing to a chirp trigger designed to stimulate a greater proportion of the microbubble population, although this did show that the short tone trigger resulted in greater release of free SN38. Examination of ultrasound triggers in vivo to improve drug delivery is justified as there are multiple mechanisms at play that may not allow direct translation from in vitro findings. In this setting, a short tone burst gives the best ultrasound parameters for tumoural drug delivery.

## 1. Introduction

Although the prognosis for patients with CRC has improved over the last two decades, the outlook in terms of disease-free survival is poor, meaning that repeated cycles of different therapeutics with debilitating side effects are required, inevitably impacting on the quality of life of patients. Many advanced drug delivery systems are being developed to deliver drugs more efficiently to solid tumours with reduced off-site toxicity. The furthest progressed of these are liposomal encapsulation of chemotherapy agents, such as Doxil^®^, which is in everyday clinical use [1]. Irinotecan is a prodrug, requiring activation by carboxylesterases to 7-Ethyl-10-hydroxy-camptothecin (SN38), its active metabolite, which is up to 1000 times more toxic. SN38 is detoxified by the polymorphic enzyme UGT1A1 to SN38 glucuronide (SN38-G). Only a fraction of the administered irinotecan dose is converted to SN38, with the remaining drug being metabolized by CYP3A4 (and possibly CYP3A5) or excreted via hepatic or renal transport [2]. Its efficacy can be further hampered by the development of neutropenia and severe delayed diarrhoea derived from enterohepatic cycling from bacterial glucuronidases producing SN38 within the gut. Therefore, SN38 has been a prime candidate for encapsulation in advanced drug delivery systems [3].

More recently, the combination of microbubbles (MBs), micron-sized gas-filled phospholipid-shelled spheres, with therapeutic liposomes has afforded an external trigger in the form of ultrasound (US) to visualise tumour vasculature entry and aid the targeted, timed release of the drug-loaded liposome. MBs volumetrically oscillate in response to the pressure waves from US and the magnitude of the oscillation is dependent on the US pressure, viscosity of surrounding medium and the gas pressure inside the MB. At <15 kPa, MB oscillation is uniform and symmetrical; however, >15 kPa to <50 kPa non-linear oscillations occur and, at >200–300 kPa, bubble collapse occurs [4]. Several mechanical phenomena arise from these oscillations that are involved in improving drug uptake into tumours. These include expansion and contraction forces on nearby cell membranes; microstreaming, where the surrounding medium moves in specific patterns in response to the oscillations and then exerts forces on nearby membranes; and micro-jetting, where the collapse of the MB can cause high-speed liquid jets to push through a membrane and shock waves can be generated from MBs reaching supersonic velocities [5,6,7]. However, despite all these phenomena being observed using ultra-high-speed camera techniques, inducing them to occur in vivo in order to improve the uptake of drugs remains more problematic. Matching the incoming US frequency to the resonance frequency of the MB population can be crucial for maximising the acoustic response, and hence therapeutic performance. This can be done either by using a mono-dispersed MB population with matched resonance [8,9], or by utilizing chirps, a coded excitation pulse that has a greater frequency range than the normal tone pulse, enabling increased excitation of polydisperse MB populations, such as those used clinically [10,11]. Research has shown that longer pulse durations or increasing the intensity of the US pulse can increase drug delivery with model drugs (mainly in vitro studies) [12,13,14].

The parameters used in vivo for drug delivery are less well studied, as may be expected. However, co-delivery of microbubbles with chemotherapy drugs is the furthest along the translational pathway. Using this method, SonoVue increased the number of treatment cycles with gemcitabine from an average of 9 to 16 cycles and two out of the five patients showed a reduction in tumour volume over the time period [15]. A follow-up of this patient group showed an almost doubled median survival time of 17.6 months compared with 8.9 months for gemcitabine treatment alone [16]. An intervening preclinical study from this group has used an increased duty cycle of 40%, which was not possible with the clinical system. This regime reduced orthotopic pancreatic tumour volumes after two treatments, although this just missed a statistically significant improvement in survival (p = 0.057) [17]. A more diverse study was recently carried out on 12 patients with liver metastases derived from primary colon cancers but two were derived from pancreatic and one from gallbladder cancers. Each received different chemotherapy treatment regimens according to their current treatment. After half an hour of chemotherapy infusion, SonoVue was injected at 4-min intervals, followed by a microbubble bursting ultrasound pulse at increasing mechanical indices (from 0.4 to 1.0). Six patients achieved stable disease, and one showed a partial response after a single treatment, although further follow-up has not yet been published [18]. In preclinical studies, Morse et al. [19] compared a 1 MHz transducer delivering a RaSP (Rapid Short Pulse) sequence or a long-pulse sequence. Both US pulse types had a peak negative pressure (P_neg_) of 0.35 MPa but RaSP was designed to deliver very stable cavitation and allow MBs to translocate. The long-pulse sequence allowed MB replenishment of the vessels in between pulses. RaSP showed enhanced dextran delivery and lower levels of damage to the brain. RaSP pulses were similarly shown to improve microbubble-mediated liposomal uptake in the brain over that obtained with long pulses [20]. The use of MBs and US was studied in 4T1 breast cancer xenografts to improve the delivery of a fluorescently labelled antibody to the tumour [21]. The total number of acoustic cycles per pulse (5000 cycles) and acoustic energy were the same in all three groups. The continuous long or mid pulses showed an improved extravasation range at the shorter time points after injection (0 and 4 h) compared to a highly fractionated short pulse.

Only one study so far has looked at the effect of different ultrasound pulses in vivo when the drug is coupled to the MB. Indocyanin green (ICG)-containing liposomes bound to MBs were used to determine the ultrasound parameters that allowed the highest deposition of ICG to mouse leg muscles without causing tissue damage [12]. Here, 5 W/cm^2^, 100% duty cycle, 2 min duration, P_neg_ of 0.39 MPa was considered optimal.

We have attempted to improve drug delivery to tumours using therapeutic microbubbles (thMBs). These microbubbles carry a chemotherapeutic payload and, in addition, are molecularly targeted to tumour regions via antibody attachment [22]. Although successful chemotherapy delivery and subsequent tumour volume reductions were achieved, we examine whether we could improve drug uptake into tumours by altering US parameters using the same thMB structure and drug each time. Alterations in the number of tone bursts within a trigger, number of US triggers and trigger type (tone or chirp) could not improve tumour drug delivery from the single 5 s tone burst trigger that we have previously used. In terms of trigger type, differences in the subsequent metabolism of the drug were observed when the trigger was changed from tone to chirp.

## 2. Materials and Methods

### 2.1. Animals

All experiments were performed following local ethical approval (University of Leeds) and in accordance with the UK Animals (Scientific Procedures) Act 1986. All experiments were conducted by project license holders with greater than 5 years’ worth experience. CD-1^®^ nude mice were bred in-house under license from Charles River Laboratories (Wilmington, MA, USA) and maintained in specific-pathogen-free conditions in individually ventilated cages (IVCs) with free access to water and food. Here, 5–7-week-old female CD-1 nude mice were used and the numbers of mice (*n*) per group are detailed in each figure legend. Mice were randomly assigned to treatment groups once tumours were approximately 6 mm × 6 mm by calliper measurement.

### 2.2. Tumour Generation

SW480 human CRC cell lines were obtained from ECACC (ecacc.org.uk, accessed on 6 March 2022) and were maintained in RPMI (Gibco, ThermoFisher Scientific, Waltham, MA, USA) with 10% (*v*/*v*) foetal calf serum (Sigma-Aldrich, St. Louis, MO, USA). All cells were maintained at 37 °C in 5% CO_2_ and cell lines were authenticated in-house by tandem repeat (STR) profiling and screened negative for mycoplasma, 5 × 10^6^ SW480 cells were injected subcutaneously to establish a xenograft on the flank of the mouse, which resulted in a 6 mm × 6 mm tumour as measured by mechanical callipers, typically by day 9, as described previously [23].

### 2.3. Therapeutic Microbubble Generation

Liposomes encapsulating SN38 were generated as previously described and freeze-dried in aliquots [22]. After reconstitution with 10 mM acetate buffer, pH 2, they were filtered through a 200 nm syringe filter. The generation and characterisation of the thMB population was carried out in a microfluidic device, as previously described [24,25]. To note, each experiment used a different preparation of SN38 liposomes encapsulating different amounts of SN38—hence, the different quantitation of SN38 by LC-MS/MS. Each experiment was internally controlled.

### 2.4. Delivery of thMBs and Ultrasound Pulses

**Figure 1:**
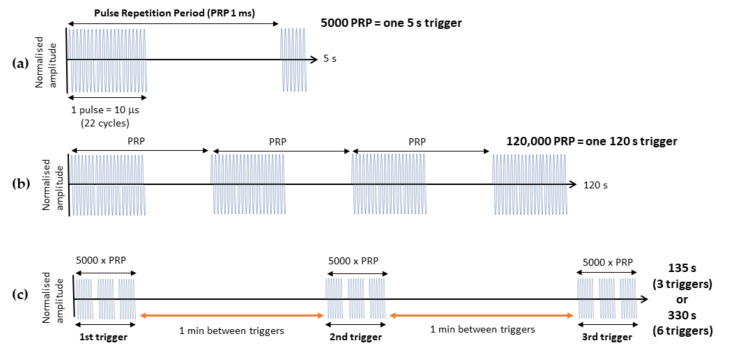
Schematic of ultrasound parameters. (**a**) One pulse consists of ~22 cycles, generated from a 2.25 MHz transducer with a pulse length of 10 μs. Since the PRF is 1 kHz, the pulse repetition period (PRP) is 1 ms, and hence with a pulse length of 10 μs, the duty cycle is 1%. A 5 s sonication time with these parameters will deliver 5000 PRPs and is termed one 5 s trigger. (**b**) Increasing the ultrasound trigger from 5 s to 120 s delivers 120,000 PRP and is termed one 120 s trigger. (**c**) Multiple triggers were created from the 5 s trigger sequence with a one min wait time between each trigger.

ThMBs (typically 1 × 10^8^ thMBs in 100–200 μL, mean diameter 1.8 μm ± 1.3 μm) were administered via tail vein injection whilst animals were awake and restrained. Animals received an US trigger at the tumour site 4 min post MB injection (unless otherwise stated) using a custom-built single element ultrasound system (UARP) [11]. A 2.2 MHz, 10 µs ‘tone burst’ US trigger was generated by an unfocused transducer (V323, Olympus NDT, Southend-on-Sea, UK), with a P_neg_ of 260 kPa, that had a 1 kHz pulse repetition frequency (PRF). The total sonication time was 5 s (Figure 1a, or expanded to 120 s in Figure 1b). Multiple triggers of 5 s sonication time were delivered with a 1-min gap in between. A chirp trigger was delivered via the same UARP with a 5 MHz unfocused transducer (V310, Olympus NDT, Southend-on-Sea, UK) producing a pulse with a P_neg_ of 110 kPa with a 1 kHz PRF. The 120 s chirp had a 6 dB bandwidth of 80% with a frequency of 3–7 MHz, and transducers were calibrated as described previously [11].

### 2.5. Tissue Processing

Thirty minutes to one hour (see figure legends for collection time point used per experiment) after injection, tumours and livers were harvested and snap-frozen in liquid nitrogen. These time points allowed drug metabolism to be measured since SN38-G indicates that SN38 has been released from the liposomes and is available for metabolism. After thawing on ice, tissues were weighed and homogenised in methanol spiked with 10 ng/mL internal standard tolbutamide (Sigma-Aldrich).

### 2.6. Mass Spectrometry for Quantitating Drug in Tissue

LC-MS/MS was carried out as previously described [22]. Multiple reaction monitoring (MRM) mass spectrometry was performed on a Waters Quattro Ultima triple quadrupole mass spectrometer with an electrospray ionisation source operating in positive ionisation mode. The peak area was generated using Masslynx software, version 4.1 (Waters Ltd., Wilmslow, UK) and compared to the standard curves of SN38 and SN38-G spiked with tolbutamide as above. The ratio of peak areas from known amounts of SN38/SN38-G to tolbutamide was plotted and used to extrapolate drug concentrations in tissues.

### 2.7. Statistical Analyses

All statistical analyses were performed using GraphPad Prism version 8 software. Statistical tests used for each experiment are described in each figure legend.

## 3. Results

### 3.1. Increasing the Ultrasound Sonication Duration

A 5 s trigger, as we have previously used [22], is very short compared to most triggers used [21,26,27,28]. Therefore, the total duration of the trigger was increased to 120 s to burst more of the injected thMB population and potentially deliver more drug to the tumour (see Figure 1 for a schematic of the US parameters). A rough theoretical framework for calculating the total amount of drug that could theoretically be delivered per US trigger was devised. Taking the volume of the tissue that will be subjected to the US and using an estimate of 1% of that volume being blood vessels, we derive the volume of vasculature that will contain MBs subjected to an US trigger. An approximation of 1% vascularity within these tumours is based on the ratio of brown pixels (CD31 stain) in a section to blue pixels (nuclei) that had a median of 0.72% IQR = 0.5–1.0 in our SW480 xenografts [22] and in other colorectal cancer xenografts [29,30]. Knowing the number of thMBs injected enables the calculation of the number of MBs within a tumour subject to the US trigger. The second parameter to calculate is the time that the US trigger is effectively acting on the MBs and is based on the duty cycle and the trigger duration. The third important parameter is the reperfusion rate, which is based on the number of heartbeats (in an awake mouse, this is around 600 bpm) and the stroke volume in these mice of ~20 μL, based on a body weight basis of 1 μL per g of mouse [31]. This allows calculation of the total volume of blood that may travel through a tumour during the US trigger. Microbubble lifetime in vivo is not significantly affected over these short time frames, as we have previously shown peak intensities in the order of several minutes [24].

Using these parameters, and an assumption that the US from the unfocused transducer used will minimally excite microbubbles to a depth of 5 mm, the volume of tissue would be 1.58 mm^3^, with 1% of this being blood vessels (0.0158 mm^3^). If the stroke volume is 20 μL and the duration of the US is 5 s, during which there are 50 heartbeats, then 1 mL of blood will be pumped around the mouse, giving a reperfusion rate of 0.2 mL/s. The concentration of the thMBs in the mouse (given an injection of 1 × 10^8^ thMBs and assuming even distribution and no sequestration), an average mouse has 58.5 mL of blood/kg [32]; therefore, a 20 g mouse has 1.17 mL of blood containing 0.85 × 10^8^ thMBs/mL. With a reperfusion rate of 0.2 mL/s and the pulse duration being 5 s, a total of 0.85 × 10^8^ thMBs would flow through the tumour vasculature during the US trigger. The first US pulse given has a 1% duty cycle; therefore, we could maximally expect 0.0085 × 10^8^ thMBs (8.5 × 10^5^ thMBs) to be burst during the US trigger over 5 s. Therefore, it is reasonable to assume that increasing the duration of the US trigger to 120 s (Figure 1b) would allow many more thMBs to be burst (2.04 × 10^7^ thMBs) and potentially deliver more drug.

**Figure 2:**
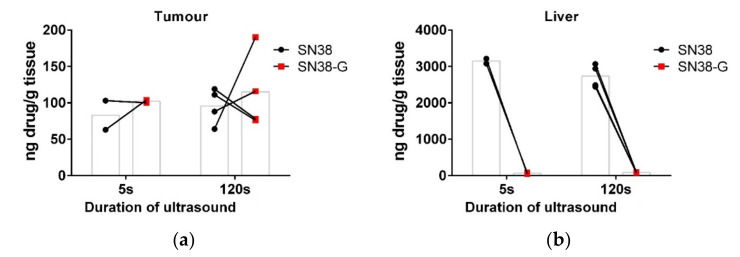
Increasing the duration of the ultrasound trigger. The effect of increasing the ultrasound trigger from 5 s to 120 s after bolus injection on drug delivery (SN38) and metabolism (SN38-G) 30 min later. Linked data points show the results from individual mice, with the bar in grey showing the mean. No difference was apparent between 5 s (*n* = 2) and 120 s (*n* = 4) for either SN38 or SN38-G in tumour (**a**) or liver (**b**).

Apart from increasing the trigger duration, all other ultrasound and MB parameters were kept the same. Tumour and tissues were taken 30 min after sonication and subject to LC-MS/MS quantitation of SN38 and SN38-G. Figure 2a shows that there was no apparent difference in measured drug delivery to the tumour following either a 5 s trigger or 120 s trigger in this small-scale study. There was also no difference in measured drug release, as shown by the glucuronidated form, which occurs when SN38 is released from the liposomes. The particle sizes used here are excreted via the hepatobiliary route; the high concentration of SN38 detected in the liver indicates the retention of the drug in the liposome, alongside the rapid excretion of any glucuronidated SN38-G from the liver due to high UGT1A1 levels. There was no apparent difference in measured drug within the liver either (Figure 2b).

### 3.2. Increasing the Number of Ultrasound Triggers

**Figure 3:**
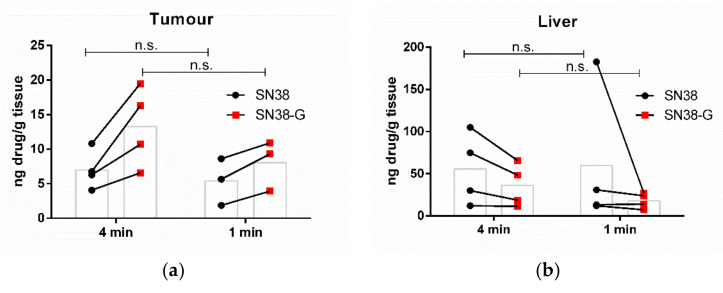

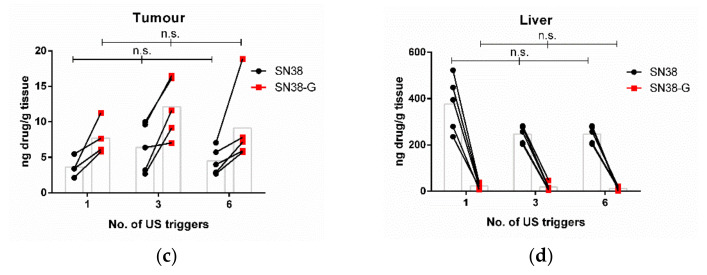
Increasing the number of ultrasound triggers. The effect of timing of the US trigger after bolus injection on drug delivery to tumour (**a**) and liver (**b**) one hour later. Linked data points show the results from individual mice, with the bar in grey showing the mean. Mann–Whitney U test showed no significant differences (*n*.s.) between 4 min (*n* = 4) and one min (*n* = 3) wait time before a single 5 s US trigger for either SN38 or SN38-G in tumour (**a**) or liver (**b**). The effect of increasing the number of 5 s US triggers on tumour drug delivery (**c**) and liver (**d**). Linked data points show the results from individual mice, with the bar in grey showing the mean. ANOVA followed by Dunn’s multiple comparisons test showed no significant differences between 1 (*n* = 4), 3 (*n* = 5) and 6 (*n* = 5) US triggers for either SN38 or SN38-G in tumour (**c**) or liver (**d**).

It is possible that a continuous pulse train as used previously might not allow optimal repopulation of the blood vessels with thMBs, potentially due to acoustic radiation forces or the PRF being shorter than the heartbeat of the mouse. Increasing the number of triggers, with wait times in between each trigger (Figure 1c), would allow replenishment of the thMB population in the tumour vasculature between triggers [4] and may therefore result in enhanced drug delivery. To optimise the balance between VEGFR2-targeted MB accumulation in the tumour vasculature and sequestration of circulating MBs by Kupffer cells, optimisation of the US trigger delay following injection was carried out. No statistically significant differences in drug delivery, biodistribution or metabolism were observed, whether the thMBs circulated for 4 min or one min before the US trigger was applied (Figure 3a,b). Hence, the effect of increasing the number of US triggers given following injection of thMB was investigated. The typical 5 s single trigger was compared to three or six 5 s triggers. thMBs were injected and 4 min were initially allowed for thMB tumour accumulation before application of the first trigger. Each subsequent trigger was applied 1 min apart, and this allowed for a shorter manual restraint of the animals and no anaesthesia required. LC-MS/MS for SN38 in the tumour and liver showed that the delivery of SN38 was not significantly improved by increasing the number of US triggers. Metabolised drug (SN38-G) indicates that the SN38 that was released from liposome encapsulation also showed no significant difference when further US triggers were used.

### 3.3. Drug Delivery and Metabolism Using a Tone Burst Trigger Versus a Chirp Trigger

**Figure 4:**
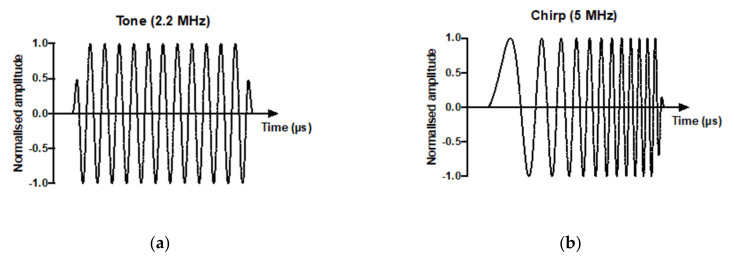

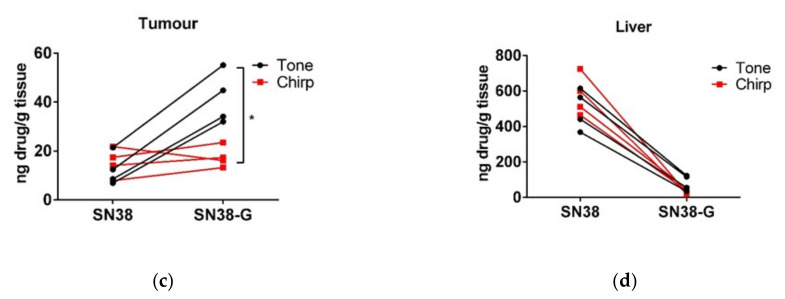
Drug delivery and metabolism using a tone burst trigger versus a chirp trigger. (**a**) Representative tone and chirp (**b**) US sequences. Concentrations of SN38 and metabolite SN38-G detected in tumours by LC-MS/MS one hour post injection of thMBs, followed by either a tone (black circles) or chirp (red squares) US triggers localised over the tumour (*n* = 4 per trigger type). Linked data points show the results from individual mice. Mann–Whitney U test showed a significant difference in the SN38-G measured in the tumour (**c**) but not the non-sonicated liver (**d**) when a tone US trigger was used (Mann–Whitney U test, two-tailed, * p = 0.029).

Since neither increasing the duration nor the number of triggers altered the efficacy of tumoural drug delivery, the use of a chirp trigger was explored. The use of a chirp trigger allows a greater proportion of microbubbles in a polydispersed population to oscillate at their resonant frequency and thus create conditions for sonoporation and potentially the release of the liposomal payload. We have previously shown this chirp trigger to improve model drug delivery in both untargeted and targeted microbubbles co-delivered with propidium iodide [11] in vitro. The 5 s tone trigger (Figure 4a) was compared to a 120 s chirp trigger (Figure 4b) and drug delivery to tumour (Figure 4c) and liver (Figure 4d) was measured one hour later.

The 5 s tone trigger showed increased metabolism of the chemotherapy delivered compared to the chirp trigger. SN38-G was found in significantly higher amounts in the tumour using a tone trigger, but no difference was found in the liver, which did not receive a direct US trigger. Therefore, a short, higher-pressure tone burst of thMBs was superior to the increased excitation of polydispersed thMBs in a population, using a chirp trigger, in terms of tumour drug delivery.

## 4. Discussion

Much work has gone into altering US parameters to improve drug delivery in vitro, with relatively little comparison at the in vivo stage. In this study, we attempted to significantly improve drug delivery to xenograft tumours by altering the US parameters but retaining the same tumour type, thMB structure and chemotherapy drug. As in other studies, we did not find any improvement in drug delivery by increasing the duration time or number of US triggers. Bush et al. [33] found no difference between a one-min and a five-min US trigger for acoustic cluster therapy. Olsman et al. [34] also found no difference with increasing US intensity. In addition, Bourn et al. [35] found no difference with an increasing number of triggers (in vitro spheroid under fluid flow). However, our experiments did show that using a short high-energy tone trigger compared to a chirp trigger resulted in greater free drug availability in the tumour, as evidenced by a statistically significant increase in metabolised drug using a tone trigger.

One current observation that may help explain the lack of further uptake is the ‘sonoprinting’ hypothesis [36]. Using high-frame rate imaging of cells and microbubble interactions, this group has shown that lipids from the surfaces of liposomes attached to MBs are deposited onto the surfaces of cells. This cell surface covering of lipids was evident only above acoustic pressures of 300 kPa and longer pulses of 100–1000 μs using a 1 MHz single element transducer. Luan et al. [37] also showed that higher pressures are required for vibration and lipid shell shedding when studying nanoparticles covalently linked to the MB shell. In our study, the US tone trigger was 260 kPa and 5000 cycles, which is very close to the regime required for sonoprinting. Since our liposome-loaded chemotherapy drug (SN38) is hydrophobic, this may result in sonoprinting of the membrane encapsulating the drug in the lipid bilayer onto the vessel wall and subsequent uptake via diffusion/endocytosis into the tumour. Therefore, further sonoprinting may not allow additional drug uptake within this time frame, due to the previous layer of lipids already deposited. Lower pressures (around 100 kPa as used in the chirp trigger) have been shown to enhance endocytosis (as described by Roovers et al. [4]), which may explain the lower levels of metabolised SN38 with the chirp trigger compared to the tone trigger. If liposomes enter the cells intact via endocytosis, the metabolism of the encapsulated drug may well be slower than a more open, sonoprinted lipid structure deposited on the surface. This may not be limited to hydrophobic drugs, as it has previously been shown in vitro that using P_neg_ of 7 MPa resulted in only around 30% of calcein release and in vivo drug uptake may well involve a significant liposomal endocytic component [38].

An alternative theory is based on earlier observations of small molecule entry into cells via co-delivery with MBs. In this scenario, pores in the cell membrane are produced through which increased endocytosis [39] or passive diffusion of small molecules can occur [40]. Therefore, it is possible that, under the conditions of our study with VEGFR2-targeted thMBs, the pore formation destroys the receptors on the membrane and so no further thMBs can bind and no further drug delivery is possible. In this case, it may take around 30 min before further drug uptake is possible. This is based on the observations that the ultrafast imaging of MBs on HUVEC cells subject to eight cycles of 1 MHz at P_neg_ 0.8 MPa showed that apical and basal cell membranes are breached and completely resealed in around 12 min [41]. FRAP experiments looking at VEGFR2 mobility showed repopulation within 8 min of photobleaching [42]; in addition, VEGFR2 is constitutively endocytosed and recycled and this process takes 20 min in HUVECs [43]. Therefore, improving drug uptake may require sequential infusions and US triggers, over a much longer time frame.

A third parameter that may have contributed to the lack of enhanced detection of drug following multiple or extended US triggers is that, from our estimation, the majority of MBs within the tumour vasculature have already been burst by the trigger during the first few microseconds. Therefore, subsequent tone triggers have far less impact on the reduced number of remaining thMBs in the circulation for the delivery of significant amounts of drug. The use of short pulse trains (e.g., 10 pulses) with a low PRF (e.g., 0.1 kHz) every 30 s may allow initial bursting of thMBs, followed by maintenance of sonoporation conditions, and allow reperfusion of the tumour vasculature with thMBs. PRFs faster than the mouse heartbeat will not allow time for full reperfusion of the blood vessels within a tumour with thMBs. It is therefore worth altering the PRF, considering the anaesthetic status of the mouse at the time of US. Longer pulse trains > 100 cycles may also result in acoustic radiation forces, aggregation and coalescence, thus shielding the thMBs from bursting and enhancing drug delivery.

## 5. Conclusions

Increasing drug delivery using US-mediated MBs can improve the on-target delivery of chemotherapies or other agents and increase the therapeutic index. Although many studies have examined US parameters in vitro, very few have studied these in vivo, where the architecture, flowrates and MB–cell interactions may well be very different. From our studies presented here, longer triggers or multiple triggers were no more effective at drug delivery to the tumour than a single, short tone burst, but this single 5 s tone burst was more effective than a chirp trigger at delivering metabolically available drug to tumours.

## Data Availability

Please contact the authors for data.
